# Dynamic transcriptomic analysis reveals suppression of PGC1**α**/ERR**α** drives perturbed myogenesis in facioscapulohumeral muscular dystrophy

**DOI:** 10.1093/hmg/ddy405

**Published:** 2018-12-06

**Authors:** Christopher R S Banerji, Maryna Panamarova, Johanna Pruller, Nicolas Figeac, Husam Hebaishi, Efthymios Fidanis, Alka Saxena, Julian Contet, Sabrina Sacconi, Simone Severini, Peter S Zammit

**Affiliations:** 1 Randall Centre for Cell and Molecular Biophysics, New Hunt's House, Guy's Campus, King's College London, London, UK; 2 Department of Computer Science, University College London, London, UK; 3 Centre of Mathematics and Physics in the Life Sciences and Experimental Biology, University College London, London, UK; 4 Genomics Research Platform, Biomedical Research Centre at Guy’s and St Thomas’ Trust and Kings College London, Guy’s Hospital, London, UK; 5 Institute for Research on Cancer and Aging of Nice, Faculty of Medicine, Université Côte d'Azur, Nice, Cedex, France; 6 Peripheral Nervous System, Muscle and ALS Department, Université Côte d'Azur, Nice, France

## Abstract

Facioscapulohumeral muscular dystrophy (FSHD) is a prevalent, incurable myopathy, linked to epigenetic derepression of D4Z4 repeats on chromosome 4q, leading to ectopic DUX4 expression. FSHD patient myoblasts have defective myogenic differentiation, forming smaller myotubes with reduced myosin content. However, molecular mechanisms driving such disrupted myogenesis in FSHD are poorly understood. We performed high-throughput morphological analysis describing FSHD and control myogenesis, revealing altered myogenic differentiation results in hypotrophic myotubes. Employing polynomial models and an empirical Bayes approach, we established eight critical time points during which human healthy and FSHD myogenesis differ. RNA-sequencing at these eight nodal time points in triplicate, provided temporal depth for a multivariate regression analysis, allowing assessment of interaction between progression of differentiation and FSHD disease status. Importantly, the unique size and structure of our data permitted identification of many novel FSHD pathomechanisms undetectable by previous approaches. For further analysis here, we selected pathways that control mitochondria: of interest considering known alterations in mitochondrial structure and function in FSHD muscle, and sensitivity of FSHD cells to oxidative stress. Notably, we identified suppression of mitochondrial biogenesis, in particular via peroxisome proliferator-activated receptor gamma coactivator 1-α (PGC1α), the cofactor and activator of oestrogen-related receptor α (ERRα). PGC1α knock-down caused hypotrophic myotubes to form from control myoblasts. Known ERRα agonists and safe food supplements biochanin A, daidzein or genistein, each rescued the hypotrophic FSHD myotube phenotype. Together our work describes transcriptomic changes in high resolution that occur during myogenesis in FSHD *ex vivo*, identifying suppression of the PGC1α-ERRα axis leading to perturbed myogenic differentiation, which can effectively be rescued by readily available food supplements.

## Introduction

Facioscapulohumeral muscular dystrophy (FSHD) is a prevalent [12/100000 (1)] skeletal myopathy, for which there is currently no cure. The condition presents most notably as a descending skeletal muscle weakness and atrophy, beginning in facial muscles (such as the orbicularis oculi and orbicularis oris), and progressing to the biceps brachii and muscles of the shoulder girdle, before affecting specific lower limb muscles such as the tibialis anterior (2,3). Interestingly, there is often a marked left–right asymmetry in the degree that muscles are affected. Curiously, muscles including the deltoids and quadriceps have less overt pathological damage until later in the disease process (2,3). In addition to myopathy, FSHD is also associated with extra-muscular features including retinal telangiectasia and sensorineural hearing loss (4–6). FSHD is highly heterogeneous, with presentations varying dramatically between first-degree relatives and even monozygotic twins (7,8). Finally, there is a differential penetrance between males and females, with males typically presenting earlier in life (9).

Genetically, FSHD is associated with loss of epigenetic repressive mechanisms including DNA methylation, histone modification and repressive chromatin proteins on an array of macrosatellite D4Z4 repeats in the subtelomere of chromosome 4q (3,10). In ∼95% of cases (FSHD1, MIM 158900), repeat-mediated epigenetic derepression occurs due to contraction of this highly polymorphic region to between 1 and 10 D4Z4 repeats (11). In the majority of the remaining ∼5% of cases (FSHD2, MIM 158901), D4Z4 epigenetic derepression is caused by mutation in the chromatin modifying gene *SMCHD1*, or in rare FSHD2 cases, by mutations in *DNMT3B* (12,13). Each D4Z4 unit encodes an open reading frame for the Double Homeobox 4 (DUX4) retrogene. Thus, epigenetic derepression allows transcription of DUX4 from the distal D4Z4 unit, which coupled with a permissive 4qA haplotype supplying a poly A signal, permits misexpression of the homeodomain-containing DUX4 transcription factor (3,11). DUX4 is normally expressed at the four-cell human embryo phase, where it activates a cleavage-stage transcriptional program (14,15). However, when ectopically expressed in FSHD, DUX4 may drive pathology by direct induction of target (e.g. pro-apoptotic) genes. This is coupled with interference by DUX4 of target gene activation by related transcription factors PAX3 and PAX7 (16,17), which could affect satellite cell-derived myoblast function during any repair/regenerative response (18).

Characterisation of FSHD patient-derived cells has revealed that FSHD myoblasts are sensitive to oxidative stress and differentiate into aberrant myotubes (16,19). Indeed, amelioration of oxidative stress in FSHD formed the basis of a recent clinical trial investigating the therapeutic potential of a cocktail of nutritional supplement antioxidants (20,21). This trial demonstrated an improvement in maximum voluntary contraction and endurance time limit of quadriceps, although showed no improvement in the 2 min walk test (20). Such results motivate investigation of other supplements that can rapidly be translated to clinic.

FSHD myotubes *in vitro* are reported to display two major phenotypes described as being smaller than control myotubes with a thin, elongated morphology and labelled as an ‘atrophic’ phenotype or myotubes of the same size as controls but displaying an unusual distribution of myonuclei and dysregulation of microtubule network, so categorised as having a ‘disorganised’ phenotype (19). Both phenotypes are currently assessed by manual inspection of immunolabelling and there is no quantitative methodology for determination of myotube size and morphology.

Proteomic studies have shown that FSHD ‘atrophic’ myotubes suppress skeletal muscle myosin heavy chain (MyHC) isoforms, whilst the ‘disorganised’ phenotype shows dysregulation of microtubule network formation, but no aberration in myosin isoforms (22). Endogenous DUX4c is more abundant in disorganised FSHD myotubes, which can be rescued by silencing DUX4c, but not DUX4 (23). It can be argued that the ‘atrophic’ myotube phenotype may be the more important contributor to the muscle weakness observed in FSHD (22,24). Therapies designed to ameliorate this phenotype could be considered likely to drive clinical improvement in patients.

While the so-called ‘atrophic’ phenotype can be induced by DUX4 (24), how this is achieved is unclear. The already barely detectable levels of DUX4 in FSHD patient biopsies and primary/immortalised muscle cultures, mean that an anti-DUX4 therapy may be insufficient (25,26). Moreover, it is important to note that whilst the term ‘atrophic’ has been used to describe the small myotubes derived from FSHD patient myoblasts, there has not been rigorous investigation as to whether they actually develop as a consequence of loss of volume from an initially larger myotube (atrophy), as opposed to reduced growth (hypotrophy) and failure to ever reach the size of control myotubes. This distinction is of importance when considering molecular pathogenesis and therapies.

Understanding how to rescue perturbed myogenic differentiation and the small FSHD myotube phenotype requires a detailed understanding of the molecular changes that occur during FSHD myogenesis in adults: a highly complex and dynamic process involving coordinated expression of a vast number of genes (27). High-frequency transcriptomic time course studies of healthy mouse and human myogenesis have revealed the importance of mechanisms that would otherwise be overlooked using fewer time points (28,29). However, such transcriptomic studies investigating FSHD myogenesis are limited, usually covering only the two time points of proliferation and terminal differentiation (30,31). Though such studies identify important molecular mechanisms in FSHD, such as HIF1α-mediated oxidative stress sensitivity (30), they are limited by lack of extensive temporal range.

Here we present a dynamic analysis of FSHD and control myogenesis. By first developing a high-throughput image analysis software, we defined a quantitative measure of the FSHD small myotube phenotype. From a panel of FSHD and control myoblast cell lines, three matched pairs for which the FSHD line was forming smaller myotubes were identified. We next characterised morphologically the most well-controlled line by high-throughput imaging of myogenesis, generating 8640 images over 5 days of differentiation in triplicate. Our imaging revealed perturbed myogenic differentiation resulting in small FSHD myotubes, which thus develop as a consequence of hypotrophy rather than atrophy. This FSHD cell line aligns and fuses more slowly than its control, allowing us to establish a set of eight critical time points in the differentiation dynamics, at each of which we performed transcriptomic investigation using RNA-sequencing, generating a total of 90 samples.

Multivariate regression analysis generated a comprehensive description of myogenesis in FSHD. Of many pathways perturbed, a clear failure was evident to activate key mediators of the mitochondrial biogenesis program during differentiation generating hypotrophic FSHD myotubes. Most notably, oestrogen-related receptor α (ERRα) and peroxisome proliferator-activated receptor gamma coactivator 1-α (PGC1α), together with their target genes, were dynamically repressed during FSHD myogenesis.

PGC1α is an essential cofactor for ERRα, an orphan nuclear receptor that up-regulates a cascade of transcription, including its own expression, to drive multiple processes including mitochondrial biogenesis (32,33). Our data indicates that suppression of PGC1α in FSHD dynamically precedes suppression of ERRα and its target genes during differentiation into hypotrophic myotubes. Crucially, siRNA-mediated knock-down of PGC1α causes the hypotrophic myotube phenotype during differentiation of control, healthy myoblasts. Moreover, supplementation with biochanin A, an isoflavone capable of increasing activity of ERRα (34), can rescue the hypotrophic myotube phenotype induced by PGC1α knock-down. Finally, biochanin A, as well as two similar acting isoflavones, daidzein and genistein, can rescue myogenic differentiation in FSHD cell lines, resulting in augmented myotube size.

## Results

### Novel, high-throughput image analysis software identifies FSHD myoblast lines with a small myotube phenotype

Myogenesis is often perturbed in FSHD, with myotube morphology variable between patient-derived differentiated myoblasts. Previous studies have categorised FSHD myotubes broadly into an ‘atrophic’ or ‘disorganised’ phenotype (19,22). Here we focus on the small or ‘atrophic’ myotube phenotype, of likely relevance to FSHD pathology. We obtained six immortalised FSHD myoblast cell lines and matched controls and one primary FSHD line and matched control. Three of these FSHD cell lines, 54-2, 54-12 and 54-A5, alongside matched control 54-6, were isolated from a biceps biopsy from a male mosaic FSHD patient (26). These lines are isogenic with the exception of the D4Z4 repeat length, which has 3 repeats in the FSHD lines, but 13 units in the control (26). Three further FSHD cell lines isolated from biceps muscle biopsies were 12Abic, 16Abic and 15Abic and sibling matched controls 12Ubic, 16Ubic and 15Ubic (35). Primary lines were derived from a male FSHD patient (MD-FSHD) and a sex-matched control (GE-CTRL). Cell lines are detailed in the Supplementary Material, Table S1.

**Figure 1:**
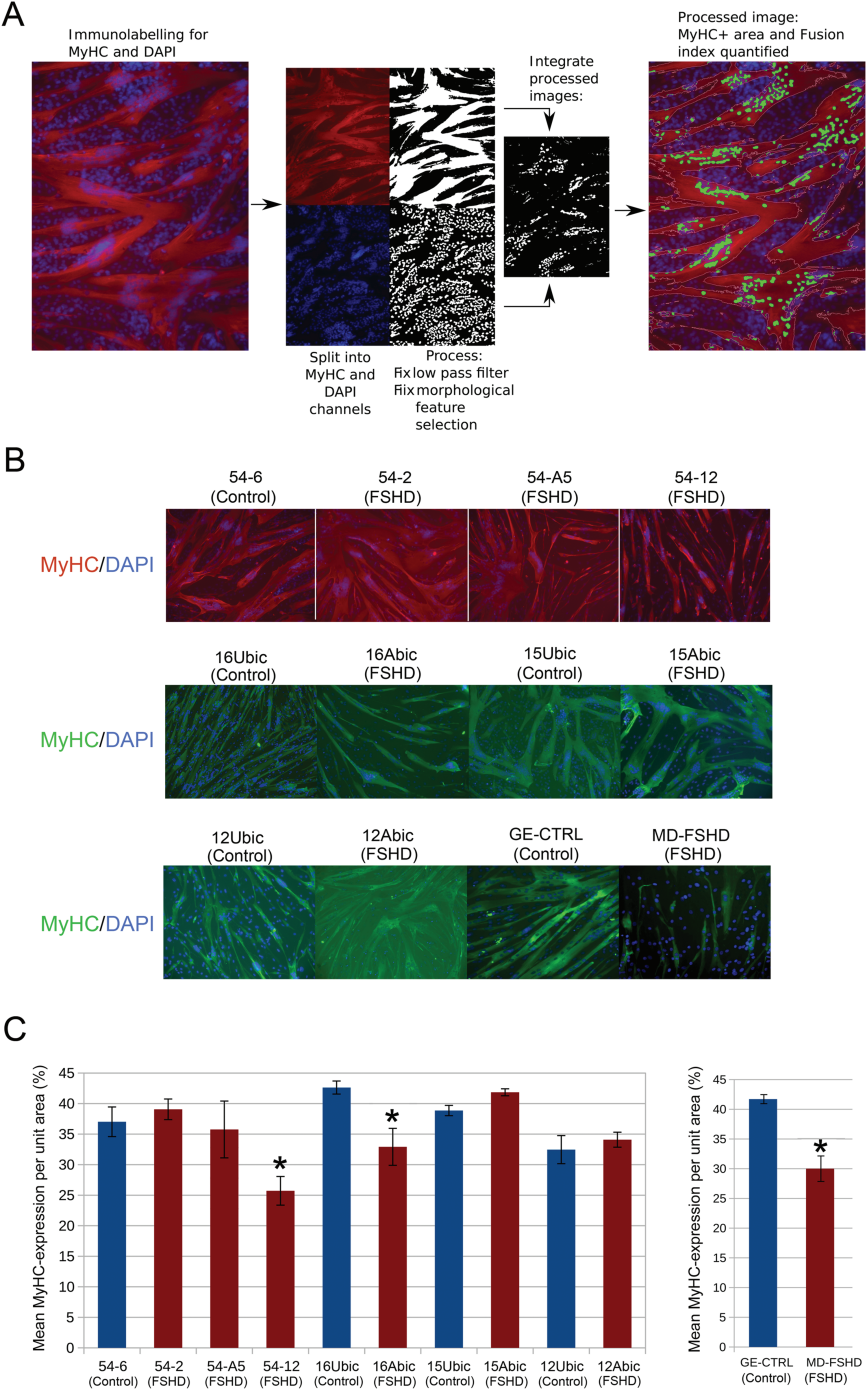
Automated image analysis demonstrates that FSHD 54-12, 16Abic and MD-FSHD cells form smaller myotubes. (**A**) Schema showing how image analysis software performs an automated image preprocessing of an MyHC immunolabelled image counterstained with DAPI and quantifies the MyHC+ve area. (**B**) Six FSHD myoblast cell lines (54-2, 54-A5, 54-12, 16Abic, 15Abic and 12Abic) and matched controls (54-6, 16Ubic, 15Ubic and 12Ubic) were plated in triplicate at 25 000 cells per well of a 96 well plate and induced to differentiate for 3 days. Primary FSHD cells MD-FSHD and controls GE-CTRL were similarly analysed. Following culture, myotubes were fixed and immunolabelled for MyHC and counterstained with DAPI to identify nuclei (Magnification: ×100). (**C**) At least three fields were imaged per well and mean MyHC+ve area was quantified from three wells/line using the automated image analysis software. FSHD 54-12, 16Abic and MD-FSHD demonstrated significantly reduced mean MyHC+ve area relative to matched controls 54-6, 16Ubic and GE-CTRL, respectively. Data is mean ± SEM (*n* = 3 wells per line), where an asterisk denotes significant difference between the MyHC+ve area in FSHD lines to matched controls (*P* < 0.05) using an unpaired two-tailed *t*-test.

These 12 cell lines were differentiated for 3 days in triplicate and immunolabelled for total MyHC using monoclonal antibody MF20. Images were processed using a novel image analysis software written using the EBImage package in R (36), to ascertain the MyHC+ve area per unit area (field) (Fig. 1A, software provided as Supplementary Material, Supplementary File 1). The FSHD 54-12 cell line relative to control 54-6, the FSHD 16Abic cell line relative to sibling control 16Ubic and the primary FSHD MD-FSHD cell line relative to control GE-CTRL clearly displayed a small myotube phenotype, defined as a reduced mean MyHC+ve area relative to matched control (Fig. 1B and C). 54-12, 54-6, MD-FSHD and GE-CTRL were derived from males whilst 16Abic and 16Ubic came from females; hence, these cell lines also permit investigation of gender-independent mechanisms of FSHD myogenesis. None of the FSHD cells lines analysed generated myotubes that were significantly bigger than their associated controls.

### Dynamic morphometric analysis of FSHD myogenesis reveals a delay in myoblast alignment and fusion

Myogenesis is a highly dynamic process involving sophisticated morphological changes culminating in cell alignment, fusion and myotube growth by both accretion of further nuclei and hypertrophy (37). The FSHD small myotube phenotype is currently defined by the appearance of thin terminally differentiated myotubes. However, it is unclear whether this is due to a defect in myotube growth or whether other morphological processes are perturbed.

**Figure 2:**
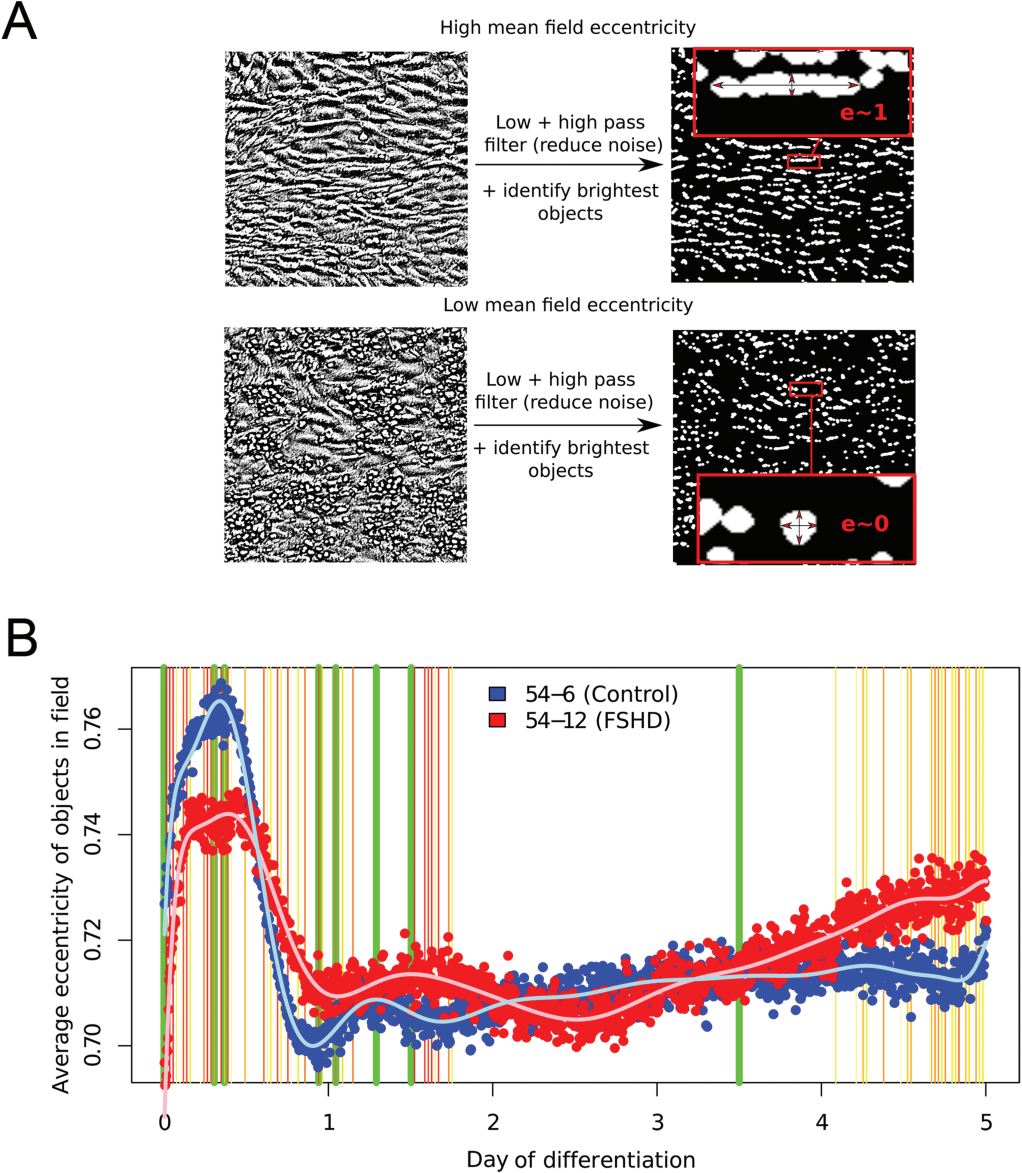
High-throughput time course imaging reveals morphological differences between myogenesis in FSHD and control myoblasts. (**A**) Schema showing how image analysis software processes and quantifies the eccentricity/elongation of cells in a phase contrast image of differentiating myoblasts. (**B**) FSHD 54-12 and matched control 54-6 myoblasts were plated in triplicate in 96 well plates and induced to differentiate over 5 days. Cells were imaged every 5 min over the differentiation process and the images processed by our software. Mean eccentricities for each cell line are plotted and a polynomial curve of best fit is shown. An empirical Bayes approach was employed to ascertain time points that showed significant differences in eccentricities control 54-6 and FSHD 54-12 cell lines. Thin vertical lines show time points that reached significance at the 5% level, and are coloured yellow to red in order of significance. Thick vertical green lines correspond to time points selected for investigation by RNA-sequencing. After day 3.5 (last vertical green line) myotubes began contracting and detaching from the plates.

To analyse morphological changes during FSHD small myotube formation compared with control myogenesis, we performed high-density, time-lapse microscopy imaging in triplicate on the isogenic (bar D4Z4 unit length) control 54-6 and FSHD 54-12 cell clones over 5 days of differentiation. One 100× phase contrast image was captured every 5 min over the process, generating a total of 8640 images (Supplementary Material, Supplementary Video 1A and B). We next developed an image analysis software to quantify morphological characteristics of each image, in particular, the mean eccentricity of the cells, which approximately corresponds to the elongation of cells (Fig. 2A). This generated a time course quantification of morphological changes during myogenic differentiation and fusion into multinucleated myotubes, which was closely reproducible across triplicates.

Control 54-6 and FSHD 54-12 followed a similar pattern of gross morphological changes, beginning with a rapid alignment phase (increasing eccentricity), followed by a cytoplasmic expansion during fusion, in which unfused cells were pushed off the plate, causing them to round up (decreasing eccentricity). Following this rapid phase of cytoplasmic expansion, the first visible myotubes appeared (increasing eccentricity, Fig. 2B). Although the gross pattern of change was similar between FSHD and control lines, the rates at which they occurred were different. An empirical Bayes approach revealed significant differences occurred particularly during the early stages of myogenesis, before the second day of differentiation (Fig. 2B). Alignment and cytoplasmic expansion phases both took longer and resulted in less extreme morphological changes in the FSHD line 54-12, as compared to the healthy line 54-6. Importantly, this imaging revealed that the small FSHD myotubes develop by failing to reach the area of control myotubes (hypotrophy) rather than losing area (atrophy).

### Dynamic transcriptomic analysis of FSHD myogenesis

Having established dynamic morphological differences between FSHD hypotrophic and control myogenesis, we next investigated the dynamic transcriptomic changes that may be driving the hypotrophic myotube FSHD phenotype. FSHD and control myoblasts fuse at different rates, with most clear morphological differences occurring before 48 h. Therefore, selection of time points for transcriptomic analysis was made to ensure that we were not only simply comparing time in differentiation, but also stage of differentiation, so that we were investigating time points where morphological defects in FSHD myoblasts are most pronounced.

Fitting polynomial curves to the average eccentricity time courses for each cell line enabled identification of three robust turning points in eccentricity for each cell line, occurring within the first 48 h of myogenesis. Examination of images corresponding to these turning points identified them as myoblast alignment, fusion and myotube growth (Supplementary Material, Fig. S1). Importantly, these events are characterised by clear morphological features, making them readily identifiable in subsequent experiments.

FSHD 54-12 and control 54-6 myoblasts were plated in triplicate and induced to differentiate. Samples were harvested for RNA-sequencing at eight time points corresponding to 0 mins (confluent proliferating myoblasts), 440 min (7.3 h, control 54-6 alignment), 530 min (8.8 h, FSHD 54-12 alignment), 1355 min (22.6 h, control 54-6 initiation of fusion), 1505 min (25.1 h, FSHD 54-12 initiation of fusion), 1860 mins (31 h, control 54-6 myotube formation), 2165 min (36.1 h, FSHD 54-12 myotube formation) and 5040 min (84 h/3.5 days, myotube maturation). Images were obtained at 100× magnification at each time point prior to harvesting, to confirm morphology matched that expected from high-throughput image analysis (Supplementary Material, Fig. S2). RNA-sequencing methodology and pre-processing are described in the Materials and Methods**.**

### Hallmarks of adult FSHD myogenesis include suppression of mitochondrial biogenesis genes regulated by ERR**α** and PGC1**α**

To assess differential expression, we employed a multivariate regression approach that exploits the temporal depth of our RNA-seq data set. Expression *E_i_* of the *i^th^* gene was modelled as a linear combination of FSHD status (cell type) and myogenic differentiation time. An interaction term was also included to investigate how FSHD status affected the relationship between gene expression and myogenic differentiation time:

$$ {E}_i={a}_i{FSHD}_{status}+{b}_i{Time}_{differentiation}+{c}_i\left({FSHD}_{status}:{Time}_{differentiation}\right) $$

Coefficient *a_i_* achieves positive values for genes whose expression is elevated in the FSHD 54-12 cell line relative to control 54-6 and negative values for genes that are repressed in FSHD 54-12 versus control 54.6. Coefficient *b_i_* achieves positive values on genes that increase their expression during myogenic differentiation and negative values on those that decrease. The interaction term coefficient *c_i_* achieves positive values on genes with expression values that increase more during differentiation in FSHD 54-12 cells than control 54-6 cells and negative values on genes with expression values that decrease more during differentiation in FSHD 54-12 cells than control 54-6 cells.

**Figure 3:**
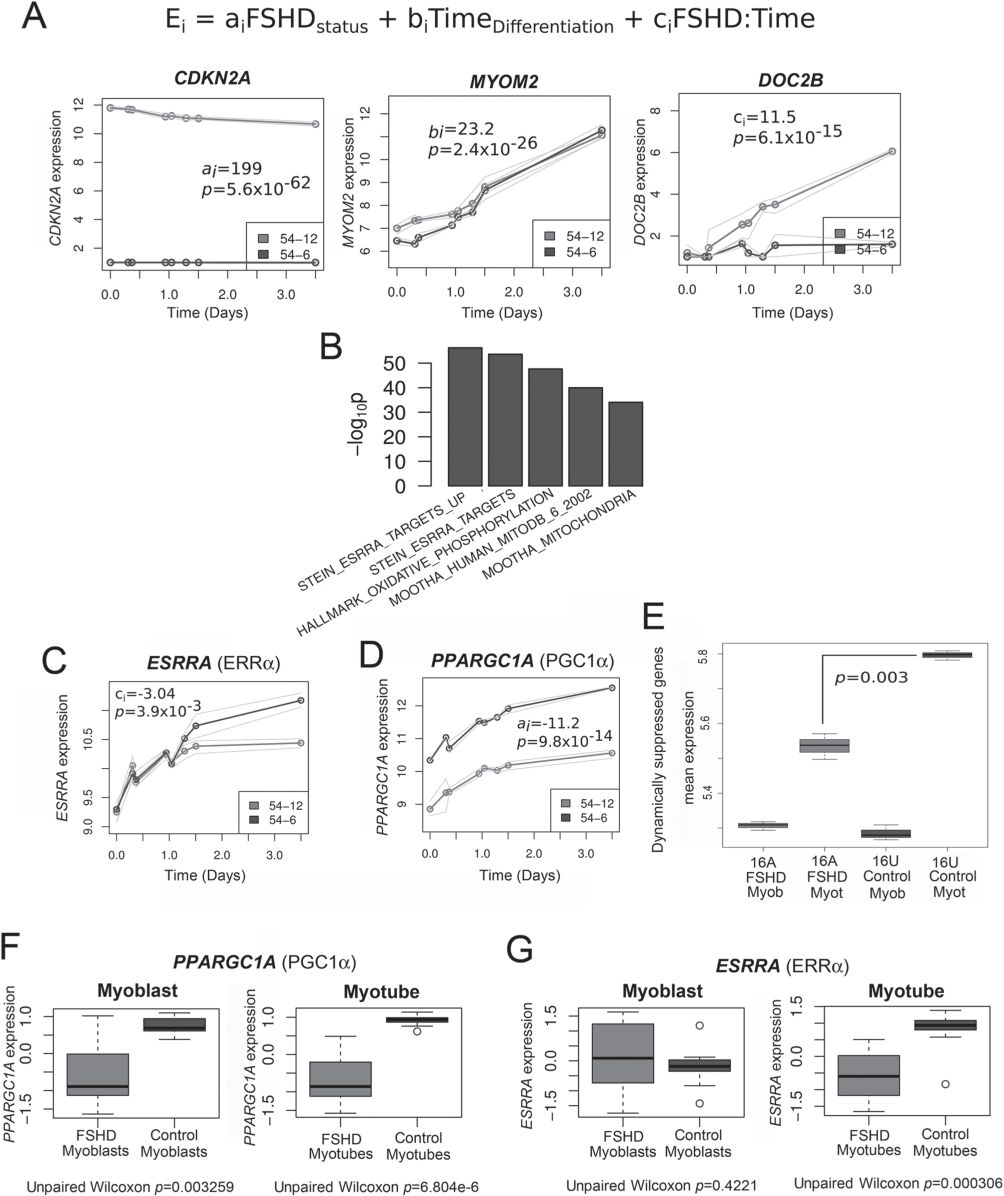
Transcriptomic analysis of FSHD myogenesis reveals suppression of PGC1α and ERRα. (**A**) A multivariate regression model was fit to the time course RNA-seq data describing the control 54-6 and FSHD 54-12 myoblasts during myogenesis. Coefficient *a_i_* attains positive values if gene *i* is up-regulated in FSHD versus controls and negative values if down-regulated. Coefficient *b_i_* attains positive values if gene *i* is up-regulated during myogenic differentiation and negative values if down-regulated. Coefficient *c_i_* attains positive values if gene *i* is up-regulated during differentiation in FSHD and negative values if down-regulated. As an example, time course expression plots are shown for the genes with the highest coefficient values for coefficient *a_i_ (CDKN2A)*, *b_i_ (MYOM2)* and *c_i_ (DOC2B)*, where thick lines represent mean expression across triplicates and thin lines denote maximum and minimum expression values observed across triplicates. (**B**) Bar plot displays log_10_ enrichment *P*-values for the top 5 enriched gene sets among the 500 genes with the most negative *c_i_* coefficient (i.e. those suppressed in FSHD myogenesis). We see clear enrichment for target genes of ERRα and genes involved in mitochondrial processes. (**C**) Expression of *ESRRA* (ERRα) in FSHD 54-12 and matched control 54-6 myoblasts from RNA-seq analysis. Significant repression in FSHD myogenesis begins from day 1 of differentiation. Thick lines represent mean expression across triplicates and thin lines denote maximum and minimum expression values observed across triplicates. (**D**) Expression of *PPARGC1A* (PGC1α) in FSHD 54-12 and matched control 54-6 myoblasts from RNA-seq analysis. Significant repression in FSHD myoblasts occurs at all time points analysed. Thick lines represent mean expression across triplicates and thin lines denote maximum and minimum expression values observed across triplicates. (**E**) The 500 genes with the most negative *c_i_* coefficient (i.e. those suppressed in FSHD myogenesis) identified in the data set of the FSHD 54-12 and control 54-6 myoblasts were tested on RNA-seq data from FSHD 16Abic and control 16Ubic at time 0, confluent myoblasts (myob) and time 5040 min, mature myotubes (Myot). Box-plots demonstrate that the mean expression of these 500 genes with the most negative *c_i_* coefficient was also significantly lower in 16Abic FSHD myotubes versus 16Ubic control myotubes. The box represents the interquartile range (IQR), with the median indicated by a line. Whiskers denote min [1.5^*^IQR, max (observed value)]; values were tested using an unpaired two-tailed *t*-test. (**F**) Expression of *PPARGC1A* (PGC1α) is suppressed in RNA-seq from FSHD 16Abic, 12Abic, 54-2 and 54-A5 cell lines at both time 0, confluent myoblasts and time 5040 min, mature myotube stage, compared to control 16Ubic, 12Ubic, 54-A10 cell lines. The box represents the IQR, with the median indicated by a line. Whiskers denote min [1.5^*^IQR, max (observed value)]; values were *z*-normalised within FSHD-control groups and tested using an unpaired Wilcoxon test. (**G**) Expression of *ESRRA* (ERRα) is suppressed only in RNA-seq from FSHD 16Abic, 12Abic, 54-2 and 54-A5 cell lines at the time 5040 min mature myotube stage, compared to control 16Ubic, 12Ubic and 54-A10 cell lines. The box represents the IQR, with the median indicated by a line. Whiskers denote min [1.5^*^IQR, max (observed value)]; values were *z*-normalised within FSHD-control groups and tested using an unpaired Wilcoxon test.

As an example, the expression pattern of the genes with the largest *a_i_*, *b_i_* and *c_i_* are shown (Fig. 3A). The gene with the largest *a_i_* coefficient was *CDKN2A,* which encodes two proteins p16, an INK4 cyclin-dependent kinase inhibitor and p14arf, an activator of p53. Up-regulation of *CDKN2A* in the FSHD 54-12 cells is consistent with reduced proliferation and increased apoptosis observed in FSHD myoblasts (19,38). The gene with the largest *b_i_* coefficient was *MYOM2,* encoding M-protein (myomesin-2), a sarcomeric protein expressed in fast skeletal muscle. The gene with the largest *c_i_* coefficient was *DOC2B*, encoding double C2 domain-β, a protein with a role in insulin sensitivity in skeletal muscle.

For each coefficient, we considered the 500 genes with the most significantly positive coefficient values and the 500 genes with the most significantly negative values and ran a Gene Set Enrichment Analysis (GSEA) (39) independently on each gene set.

For coefficient *a_i_*, genes associated with positive values are up-regulated in the FSHD cell line. These were highly enriched for a number of stem cell gene sets, in line with our recent work demonstrating that DUX4 induces a less differentiated transcriptome (38; Supplementary Material, Table S2). Genes negatively associated with the FSHD cell line were highly enriched for multiple components of the polycomb repressive complex 2 (PRC2) and H3K27me3 (Supplementary Material, Table S3), suggesting that this mode of epigenetic repression is lost in FSHD, consistent with a number of studies by ourselves and others suggesting epigenetic derepression in FSHD (40–43).

For coefficient *b_i_,* genes associated with positive values are those that increase during differentiation regardless of cell type. As anticipated, these included many troponins, actins and myosins. The most significantly enriched gene set for these genes identified by GSEA was the HALLMARK_MYOGENESIS gene set (*P* = 3×10^-51^; Supplementary Material, Table S4), a gene set collated by the Broad Institute (39) containing genes known to be associated with skeletal muscle myogenesis. Its strong enrichment here confirms our data set of myogenic differentiation shows concordance with other studies (39). Genes associated with negative values of *b_i_* (i.e. those suppressed in myogenesis) were significantly enriched for proliferation gene sets and genes involved in promoting oligodendrocyte differentiation (Supplementary Material, Table S5).

For coefficient *c_i_,* genes associated with positive values are those that are enhanced more during myogenic differentiation in FSHD. (Supplementary Material, Table S6). These genes were highly enriched for targets of LEF1, a downstream component of canonical Wnt/β-catenin signalling that we and the others identified as aberrantly active in FSHD muscle (5,43,44). Transcriptional targets of MYOD1 were also significantly enriched, indicating inappropriate activation of MYOD1 during myogenesis in the FSHD cell line, in line with previous findings (16,45).

Importantly, genes associated with negative values of the interaction term coefficient *c_i_* are those repressed specifically in FSHD differentiation (Supplementary Material, Table S7). Here we see a strong and consistent enrichment for ERRα transcriptional target genes, as well as mitochondrial genes, oxidative phosphorylation, the tricarboxylic acid cycle (TCA) cycle and PGC1α target genes (Fig. 3B).

### Suppression of mitochondrial biogenesis genes regulated by ERR**α** and PGC1**α** is a general feature of FSHD myogenesis

Of the many pathways that we found perturbed during myogenesis in FSHD (Supplementary Material, Tables S2**–**7), we decided to focus on the ERRα/PGC1α pathway, of interest considering the sensitivity of FSHD cells to oxidative stress and know mitochondrial dysfunction (21,45,46). Further examination revealed that in addition to insufficient activation of PGC1α/ERRα target genes, there was also significant suppression of ERRα transcripts (encoded by *ESRRA*) in differentiating FSHD 54-12 myoblasts, beginning from around 24 h of differentiation (Fig. 3C). PGC1α transcripts (encoded by *PPARGC1A*) were significantly suppressed at all time points of differentiation in the 54-12 FSHD myoblasts compared with 54-6 controls (Fig. 3D).

To validate our findings from the FSHD 54-12 myogenesis, further RNA-sequencing was performed on FSHD myoblast cell lines 16Abic, 12ABic, 54-2 and 54-A5, alongside matched control lines 16Ubic, 12UBic and 54-A10, in triplicate at 0 min (confluent proliferating myoblasts) and 5040 min (84 h/3.5 days, mature myotubes) of differentiation. Of the comprehensive description of FSHD myogenesis revealed by our analysis of the transcriptomics data, we were particularly interested in the 500 genes with the most significant negative associations with the interaction term coefficient *c_i_*. In line with the derivation of these genes as those insufficiently activated during FSHD myogenesis, we found that while they showed similar levels in FSHD 16Abic and control 16Ubic confluent myoblasts, the level of these genes was significantly lower in mature FSHD 16Abic myotubes, as compared with control 16Ubic myotubes (*P* = 0.003, Fig. 3E). We next evaluated expression of *ERRα* and *PGC1*α transcripts in FSHD 16Abic, 12Abic, 54-2 and 54-A5 cell lines or control 16Ubic, 12Ubic, 54-A10 cell lines at myoblast and myotube stage. Consistent with our findings in the 54-12 and 54-6 cell lines, *PGC1α* was suppressed in both combined FSHD myoblasts (*P* = 0.0033, Fig. 3F) and myotubes (*P* = 6.8×10^-6^, Fig. 3F) relative to combined controls, whilst *ERRα* was only suppressed in combined FSHD myotubes (*P* = 0.00031, Fig. 3G).

### Knock-down of PGC1**α** recreates the hypotrophic FSHD myotube phenotype in control myoblasts

Our dynamic transcriptomic analysis implicates suppression of PGC1α leading to insufficient activation of ERRα, as a critical molecular mechanism underlying perturbed differentiation and the FSHD hypotrophic phenotype. Given this model, we next investigated whether suppression of PGC1α can drive formation of a hypotrophic myotube phenotype in control myoblasts.

**Figure 4:**
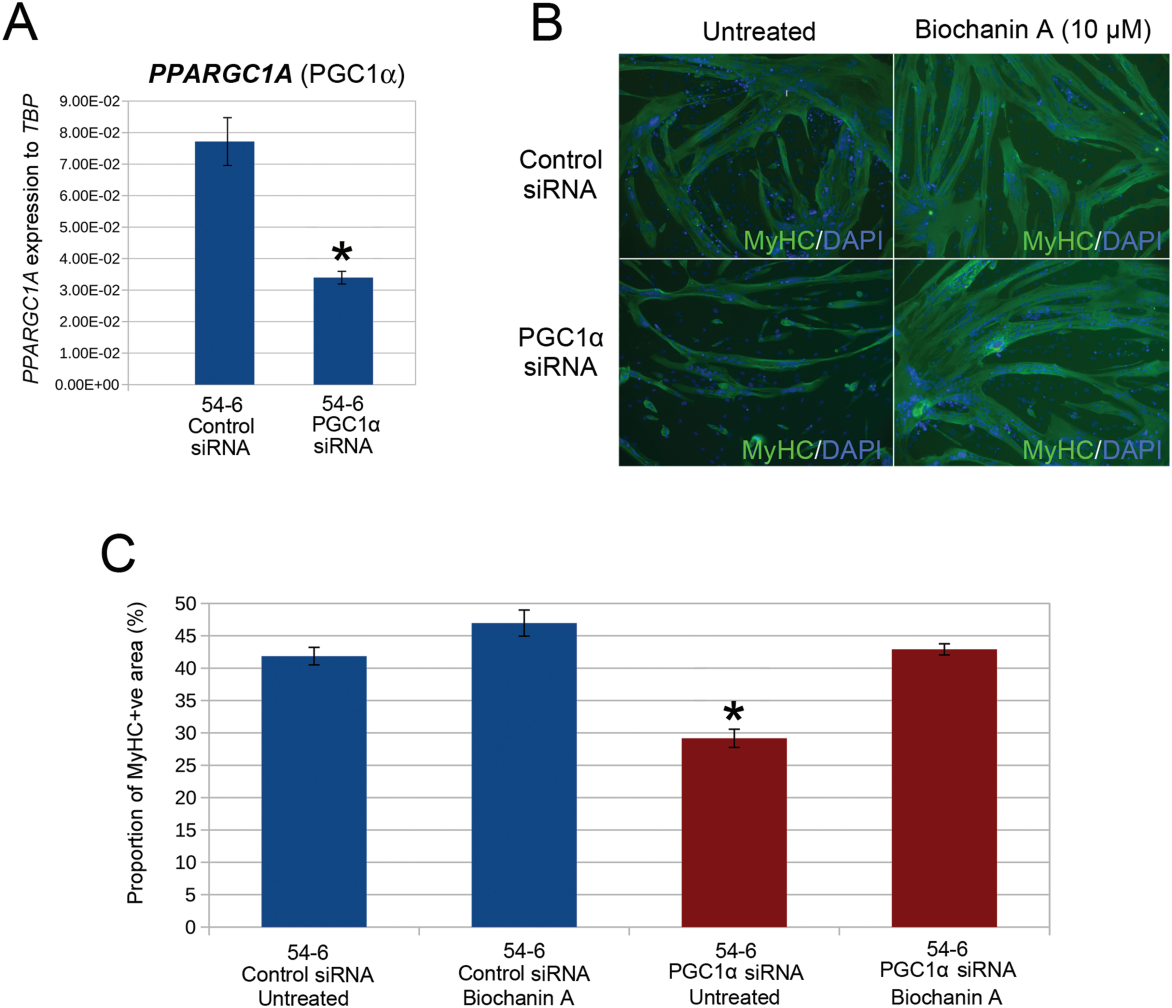
siRNA-mediated knock-down of PGC1α is sufficient to cause the hypotrophic FSHD myotube phenotype, which can be rescued by the ERRα agonist biochanin A. (**A**) RT-qPCR demonstrates that four combined siRNAs against PGC1α successfully suppresses PGC1α (*PPARGC1A*) in control 54-6 myoblasts. Data expressed as mean ± SEM where an asterisk denotes significant difference (*P* < 0.05) using an unpaired two-tailed *t*-test. (**B**) Control 54-6 myoblasts were transfected with a mixture of four siRNAs against PGC1α or a scrambled siRNA control and induced to differentiate for 3 days. Control 54-6 myoblasts were also transfected with combined siRNAs against PGC1α or a scrambled siRNA control but also exposed to 10 μm biochanin A during 3 days of differentiation. Myotubes were then immunolabelled for MyHC and all nuclei counterstained with DAPI (Magnification: ×100). (**C**) PGC1α knock-down significantly reduced MyHC+ve area. However, this PGC1α knock-down mediated reduction in MyHC+ve area could be rescued to control levels by administration of 10 μm biochanin A to the differentiation medium. Data expressed as mean ± SEM (*n* = 3 wells per line) where an asterisk denotes significant difference between the MyHC+ve area in 54-6 control siRNA/untreated versus treated conditions (*P* < 0.05) using an unpaired two-tailed *t*-test.

Control 54-6 myoblasts were plated in triplicate and co-transfected with either a mixture of four independent siRNAs against PGC1α or scrambled control siRNAs. Effective siRNA-mediated knock-down of PGC1α was confirmed by RT-qPCR (Fig. 4A). Transfected cells were then induced to differentiate for 3 days, before fixation and immunolabelling for MyHC (Fig. 4B). Cells were then imaged and MyHC+ve area quantified using our image analysis software (Fig. 4C).

Control 54-6 myoblasts transfected with siRNA against PGC1α and then subjected to a differentiation protocol had a significantly lower mean MyHC+ve area and displayed a hypotrophic myotube phenotype on day 3 of differentiation, as compared to controls transfected with scrambled siRNA (Fig. 4B and C). This demonstrates that suppression of PGC1α, as observed in FSHD myoblasts, is sufficient to cause a hypotrophic phenotype in control myoblasts.

### ERR**α** agonist biochanin A rescues the hypotrophic myotube phenotype caused by knock-down of PGC1**α** in control myoblasts

PGC1α is a critical cofactor of ERRα, an orphan nuclear receptor that orchestrates a transcriptomic program regulating mitochondrial biogenesis and other processes. As we observe insufficient activation of ERRα and its related mitochondrial biogenesis-associated target genes in FSHD myoblasts during differentiation into hypotrophic myotubes, it is likely that PGC1α is driving the hypotrophic myotube phenotype through an insufficient activation of *ERRα*. We thus postulated that activation of ERRα in a PGC1α-independent manner might rescue the hypotrophic myotube phenotype caused by PGC1α knock-down.

**Figure 5:**
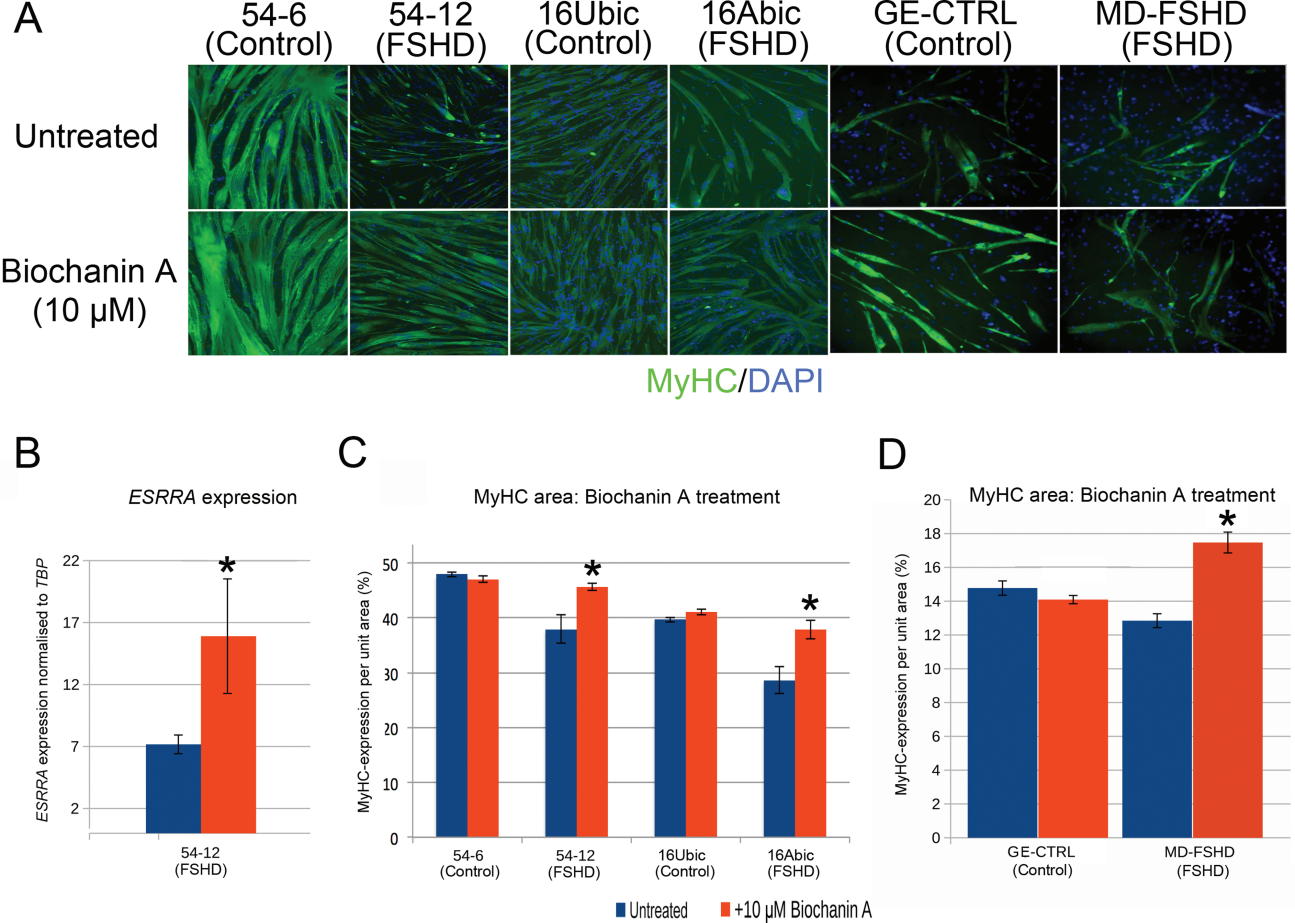
ERRα agonist biochanin A rescues the FSHD hypotrophic myotube phenotype. (**A**) FSHD 54-12 and 16Abic myoblast lines and primary MD-FSHD alongside matched controls 54-6, 16Ubic and GE-CTRL were induced to differentiate with/without 10 μm biochanin A for 3 days, fixed and immunolabelled for MyHC and nuclei counterstained with DAPI (Magnification: ×100). (**B**) RT-qPCR demonstrates that 10 μm biochanin A significantly increases expression of *ESRRA* in FSHD 54-12 myotubes. Data expressed as mean ± SEM (*n* = 3 wells per line) where an asterisk denotes significant difference (*P* < 0.05) using an unpaired two-tailed *t*-test. (**C** and **D**) FSHD myoblast lines 54-12 and 16Abic and primary FSHD cells MD-FSHD alongside matched controls 54-6, 16Ubic and GE-CTRL were induced to differentiate with/without 10 μm biochanin A for 3 days, fixed, immunolabelled for MyHC and MyHC+ve area quantified. All three FSHD cell lines demonstrated increased MyHC+ve area with biochanin A, whilst control myotubes were unaffected. Data expressed as mean ± SEM (*n* = 3–5 wells per line), where an asterisk denotes significant difference between the MyHC+ve area in untreated versus biochanin A treated myotubes (*P* < 0.05) using an unpaired two-tailed *t*-test.

Biochanin A is an isoflavone found in red clover extract and soy that binds and activates ERRα directly (34) and has an excellent safety profile in clinical trials (47,48). Administration of 10 μm biochanin A to the differentiation medium was sufficient to rescue to control levels the hypotrophic myotube phenotype induced by PGC1α knock-down in control 54-6 myoblasts (Fig. 4B and C). Interestingly, biochanin A had no effect on mean MyHC+ve area in myoblasts transfected with control scrambled siRNA, indicating that the capacity of biochanin A to counter the hypotrophic myotube phenotype is specific to suppression of the PGC1α-ERRα axis (Fig. 4B and C).

### Biochanin A, daidzein or genistein rescue the hypotrophic myotube phenotype in FSHD

We next investigated whether biochanin A can reduce the hypotrophic myotube phenotype in FSHD myoblasts, where PGC1α is endogenously suppressed (Fig. 3). We plated FSHD myoblasts that give hypotrophic myotubes, namely FSHD 54-12, 16Abic and MD-FSHD myoblasts alongside control 54-6, 16Ubic and GE-CTRL myoblasts (Fig. 1) and induced differentiation with/without 10 μm biochanin A. After 3 days, cells were fixed and immunolabelled for MyHC, images were acquired and analysed for MyHC+ve area (Fig. 5A). To first check that biochanin A was targeting ERRα, RNA was isolated from FSHD 54-12 myotubes and RT-qPCR performed, which confirmed a significant mean 2.3 fold up-regulation of ERRα (which auto-activates) in biochanin A-treated samples (Fig. 5B). Biochanin A significantly increased mean MyHC+ve area of myotubes in all three FSHD cell lines (Fig. 5C and D). However, biochanin A had no effect on average MyHC+ve area in control 54-6, 16Ubic and GE-CTRL myotubes (Fig. 5C and D).

**Figure 6:**
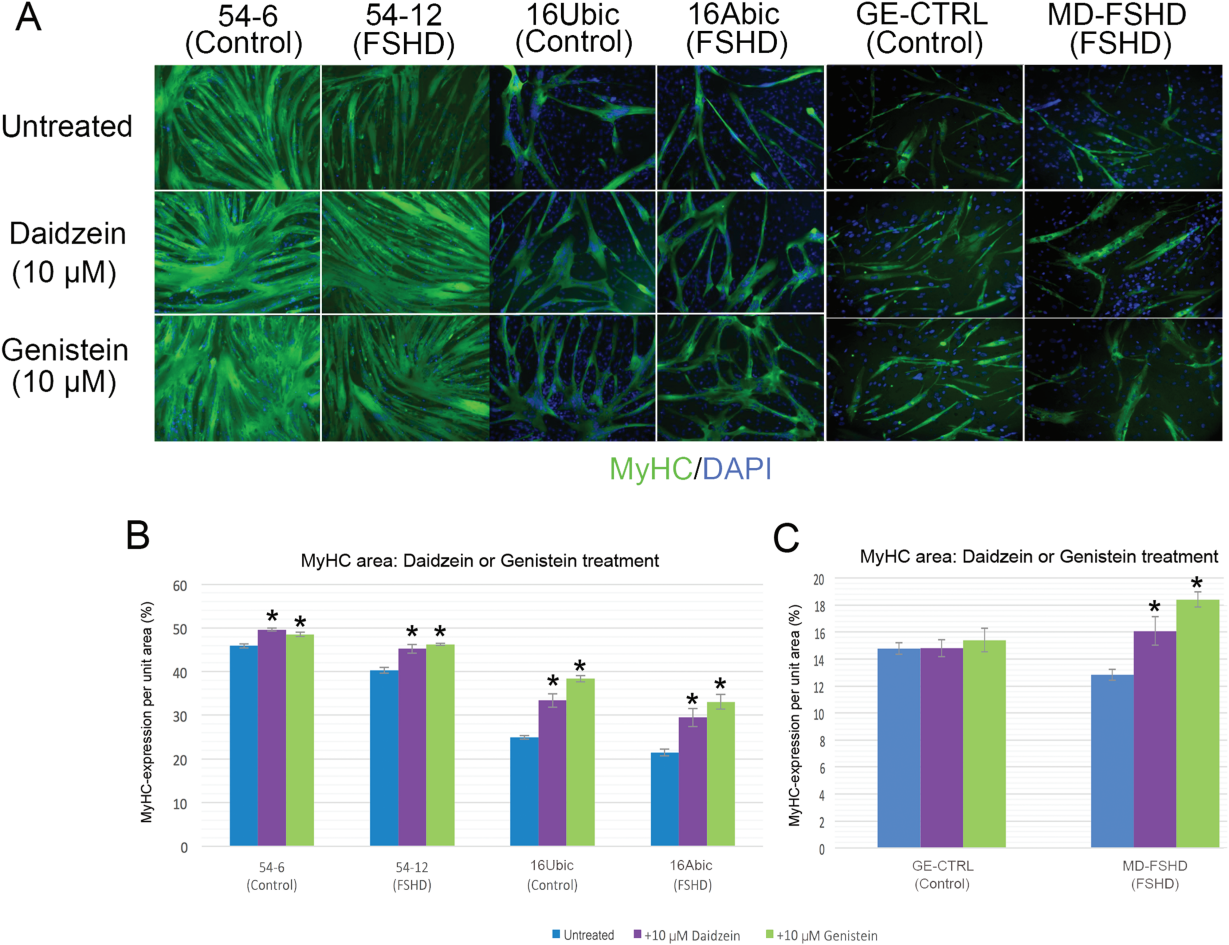
ERRα agonists daidzein or genistein rescue the FSHD hypotrophic myotube phenotype. (**A**) FSHD myoblast lines 54-12 and 16Abic and primary FSHD cells MD-FSHD alongside matched controls 54-6, 16Ubic and GE-CTRL were induced to differentiate with/without 10 μm daidzein or 10 μm genistein for 3 days, myotubes were then fixed, immunolabelled for MyHC and nuclei counterstained with DAPI (Magnification: ×100). (**B**–**C**) Quantifying MyHC+ve area showed that daidzein or genistein increased MyHC+ area in myotubes of all FSHD cell lines, as well as in myotubes of the control 54-6 and 16Ubic lines, but not in primary control myotubes. Data expressed as mean ± SEM (*n* = 3 wells per line) where an asterisk denotes significant difference from untreated (*P* < 0.05) using an unpaired two-tailed *t*-test.

**Figure 7:**
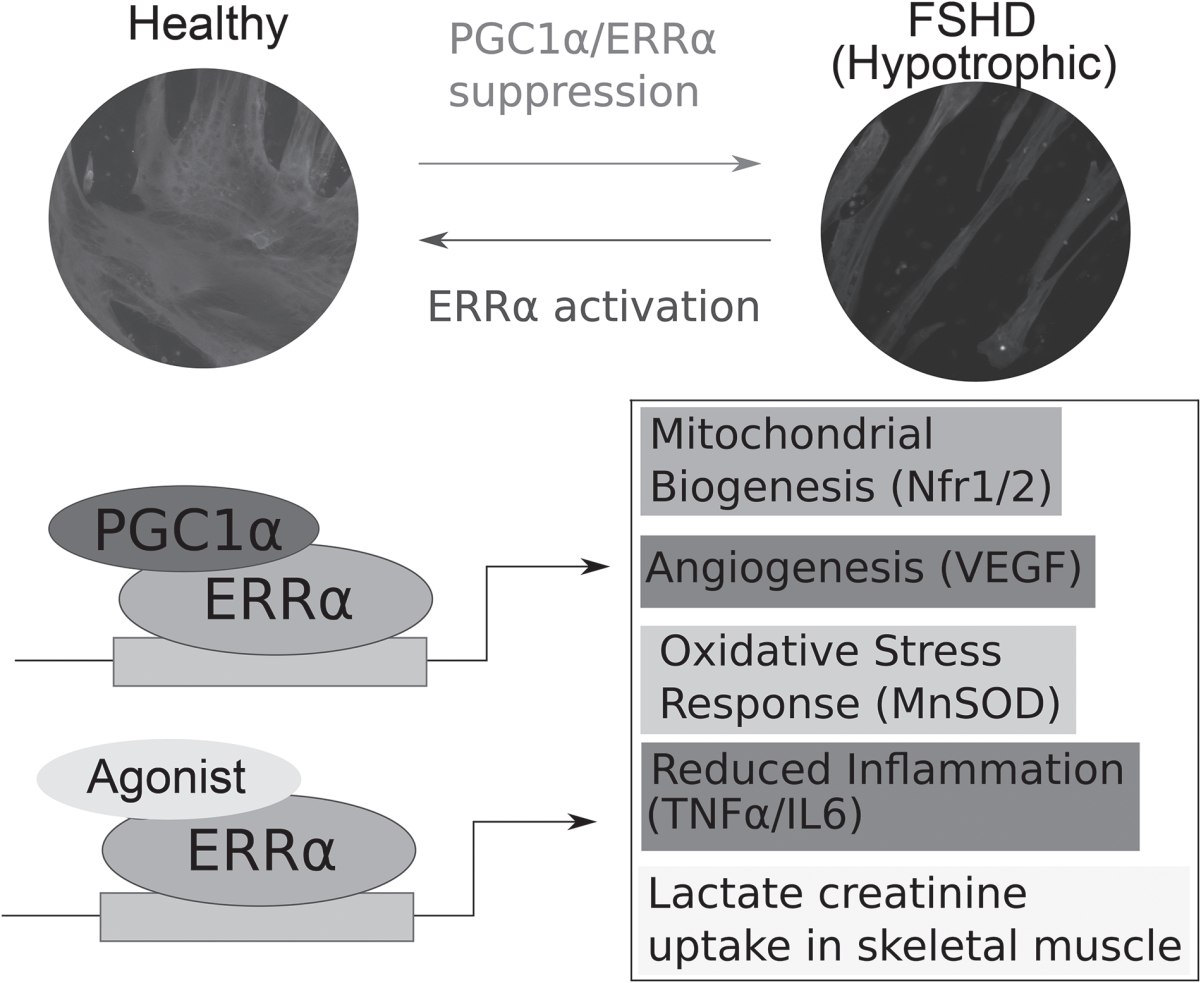
Suppression of *PGC1*α/*ERR*α expression in FSHD and rescue by ERRα agonists. Schematic summarising that PGC1α/ERRα suppression in FSHD drives an FSHD hypotrophic phenotype that can be rescued by ERRα agonists biochanin A, daidzein or genistein. Suppression of the ERRα/PGC1α pathway in FSHD patients could also contribute to know features of FSHD pathology including oxidative stress sensitivity, aberrant vasculature and inflammation.

Daidzein and genistein are isoflavones that have also been demonstrated to activate ERRα*in vitro*, although to lower levels than biochanin A (34). We differentiated FSHD 54-12, 16Abic and MD-FSHD myoblasts alongside control 54-6, 16Ubic and GE-CTRL with/without 10 μm daidzein or 10 μm genistein. After 3 days, myotubes were fixed and immunolabelled for MyHC, images were acquired and analysed for MyHC+ve area (Fig. 6A). Consistent with observations obtained with biochanin A, daidzein or genistein both increased mean MyHC+ve area in the FSHD cell lines (Fig. 6B and C). However, daidzein or genistein also acted to increase mean MyHC+ve area in control 54-6 and 16Ubic lines, but not in the primary control GE-CTRL (Fig. 6B and C). Thus, ERRα agonists biochanin A, daidzein or genistein can rescue the perturbed myogenesis that leads to formation of FSHD hypotrophic myotubes (summarised in Fig. 7).

## Discussion

Here we coupled high-throughput time course imaging and transcriptomics, generating over 8640 images and 90 RNA-seq samples of healthy and FSHD myogenesis, to provide the first dynamic analysis of FSHD differentiation and myotube formation. Of the many transcriptional changes identified in this comprehensive description of myogenesis in FSHD, we concentrated on the finding that suppression of PGC1α leads to a dynamic repression of ERRα from day 1 of differentiation, driving FSHD hypotrophic myotube formation. PGC1α knock-down is sufficient to create a hypotrophic myotube phenotype in control myoblasts. Importantly, FSHD myogenesis and hypotrophic myotubes can be rescued by administration of ERRα agonists biochanin A, daidzein or genistein (Fig. 7).

A general caveat of this study is that the degree of myotube maturation attainable *ex vivo* is limited, so we are focusing on myogenic differentiation and the early phases of myotube maturation, but such stages are relevant for understanding the repair/regeneration response in FSHD and for designing regenerative therapies. Our examination and measurement of myogenesis indicates that the term ‘atrophic’ used to describe FSHD myotubes with a thin morphology (17) is misleading, as the myotubes do not appear to lose volume. Rather, these FSHD myotubes never attain the volume of controls, so a better description is a ‘hypotrophic’ phenotype, as adopted here. While selecting for FSHD myoblast lines that give hypotrophic myotubes may bias our analysis, a general reduction in muscle fibre cross-sectional area is characteristic of FSHD muscle biopsies. Many muscle fibres clearly show an atrophic phenotype (49). However, it can be argued that a failure of regenerating muscle fibres to reach the size of mature muscle fibres may also contribute to such muscle fibre size variability, which we can model using hypotrophic myotubes.

PGC1α is a critical component of the mitochondrial biogenesis pathway that it initiates via ERRα activation in two ways; firstly, it induces expression of ERRα, and secondly, it directly interacts with ERRα to increase its ability to activate transcription, which is minimal in the absence of PGC1α (50). Myoblasts isolated from FSHD patients display an oxidative stress sensitivity phenotype, and a number of molecular mechanisms have been proposed to underlie this, including over-activation of HIF1α signalling (30,43), glutathione redox pathway dysregulation (16) and importantly, mitochondrial dysfunction (21). Mitochondria in FSHD display aberrant ultrastructure and distribution within the myofibre, as well as defects in cytochrome c oxidase activity and adenosine triphosphate (ATP) synthesis via the oxidative phosphorylation (OXPHOS) pathway (21). Moreover, these mitochondrial deficits correlate with functional muscle impairment in FSHD (21). A molecular understanding of mitochondrial dysfunction in FSHD remains elusive. However, it is likely that the suppression of PGC1α that we report, here could play a crucial role.

FSHD patients also display a decreased ratio of reduced glutathione (GSH) to oxidized glutathione (GSSG), leading to a sensitivity to oxidative stress (21). PGC1α knockout causes a decrease of GSH, resulting in a reduced reactive oxygen species (ROS) detoxification (51). PGC1α is also directly involved in defence against oxidative stress by the up-regulation of antioxidant enzymes such as Manganese superoxide dismutase (MnSOD), and increased levels of PGC1α have been shown to reduce damage attributed to ROS (52,53). FSHD and DUX4 expressing myoblasts produce higher levels of ROS than matched controls (46) and such ROS accumulation may be attributable to mitochondrial dysfunction. Suppression of PGC1α in FSHD may inhibit the antioxidant response to this elevated ROS. Crucially, a key target gene of PGC1α is MnSOD, which is an antioxidant enzyme not found up-regulated in FSHD muscle (21).

In addition to up-regulation of mitochondrial biogenesis and suppression of oxidative stress, the PGC1α/ERRα complex also acts to induce angiogenesis by up-regulating Vascular endothelial growth factor (VEGF) in a HIF-1 independent manner (54). This latter mechanism has been linked to poor/aberrant vascularisation of skeletal muscle and the retina when PGC1α is suppressed (54,55). As well as muscle atrophy in FSHD, there are well-reported vascular defects including retinal telangiectasia (4,5) and a reduction in skeletal muscle capillary density (56). Both of these vascular abnormalities could be explained by a suppression of PGC1α in FSHD.

Moreover, PGC1α suppression also results in a low-grade inflammation and an up-regulation in serum TNFα and IL6 (57). FSHD skeletal muscle has increased T-cell infiltration (58) and patients display elevated serum TNFα in a manner that negatively correlates with muscle function (21). Lastly, PGC1α suppression has been previously implicated in muscle atrophy, both during sarcopenia and in muscle wasting in chronic disease, whilst up-regulation of PGC1α has been associated with resistance to muscle atrophy (59,60).

PGC1α can be induced by a number of mechanisms, including cold temperature and exercise, both of which are mediated by β_2_-adrenergic activity (61). It is of note that five clinical trials have investigated β_2_ agonists (albutamol/salbutamol) in FSHD. Though these trials did not report significant improvements in primary outcome measures, and treated groups experienced adrenergic side effects, three of the trials reported improvements in secondary measures including lean body mass and muscle volume (62–66). We have shown that suppression of PGC1α may drive muscle hypotrophy in FSHD, it is possible that the reported increases in muscle mass under β_2_-adrenergic stimulation may be driven by up-regulation of PGC1α.

A more recent clinical trial has investigated the role of antioxidant dietary supplements in FSHD including vitamin C, vitamin E, selenium and zinc, which were well tolerated with no associated side effects (20). The study reported modest improvements in maximal voluntary contraction of quadriceps but no improvement in 2 min walk test. Selection of supplements, however, was not targeted to any particular molecular mechanism and one may anticipate stronger results if this were the case. Here we have shown that safe ERRα agonists biochanin A, daidzein or genistein can rescue the hypotrophic myotube phenotype when FSHD myoblasts differentiate. Given the molecular motivation of these molecules and their safety profiles, they could be considered alongside antioxidants in future FSHD clinical studies.

In summary, we have comprehensively described the morphological and allied transcriptional changes that occur during myogenesis in FSHD. Of particular note, such dynamic analysis of high-throughput data revealed that PGC1α suppression leading to ERRα repression in FSHD contributes to perturbed myogenic differentiation and hypotrophic myotube formation. Moreover, modulation of the PGC1α-ERRα pathway by nutritional supplements such as biochanin A, daidzein or genistein may prove a rapidly translatable therapeutic approach for improving muscle condition and repair/regeneration in FSHD patients.

## Materials and Methods

### Cell culture and myogenic differentiation

Three immortalised FSHD human myoblast cell lines 54-12, 54-A5, 54-2 (3 D4Z4 units) together with two control lines 54-6 and 54-A10 (13 D4Z4 repeats), all from the biceps of a mosaic FSHD1 patient (26), were kind gifts from Dr Vincent Mouly of the Center for Research in Myology, Paris. Six immortalised human myoblast lines from the biceps of three FSHD1 patients, 12Abic, 15Abic and 16Abic and three sibling-matched controls 12Ubic, 15Ubic and 16Ubic (33), were kind gifts from Professor Charles Emerson from the UMMS Wellstone Centre for FSHD. We isolated primary myoblast cell line MD-FSHD from the quadriceps of a 27-year-old FSHD1 patient with seven D4Z4 repeats, while control GE-CTRL was isolated from the quadriceps of an unrelated 48-year-old male. Briefly, fresh biopsies were digested with Liberase™ TM (Research Grade) and cells amplified to around 10 million, before being immunolabelled using Mouse Anti-Human CD56 (Clone B159, BD Bioscience, CA, USA), then sorted via Fluorescence-activated cell sorting (FACS) (ARIA III) and further cultured to P5 (Supplementary Material, Table S1).

Human myoblasts were cultured in Skeletal Muscle Cell Growth Medium (Promocell via VWR International Ltd, Leicestershire, UK) supplemented with 20% foetal bovine serum (ThermoFisher Scientific, MA, USA), 50 μg/ml fetuin (bovine), 10 ng/ml epidermal growth factor (recombinant human), 1 ng/ml basic fibroblast growth factor (recombinant human), 10 μg/ml insulin (recombinant human), 0.4 μg/ml dexamethasone and 50 μg/ml gentamycin, at 37^o^C under 5% CO_2_. To induce differentiation, myoblasts were washed with phosphate buffered saline (PBS) and placed in DMEM Glutamax (ThermoFisher Scientific) supplemented with 1/1000 recombinant bovine insulin (Sigma-Aldrich, Dorset, UK) and 1/1000 gentamycin (ThermoFisher Scientific) at 37^o^C under 5% CO_2_. 10 μm biochanin A (Cayman Chemical Company, MI, USA), daidzein (Cayman Chemical Company) or genistein (FluoroChem, Derbyshire, UK) were added to the differentiation medium as indicated. Biochanin A stock solution was dissolved in water, so control samples were not supplemented with any excipient. Daidzein or genistein stock solution was dissolved in dimethyl sulfoxide (DMSO) and an equivalent concentration of DMSO was added to controls. Primary human myoblasts were treated with all drugs dissolved in DMSO and an equivalent concentration of DMSO added to controls.

### RNA-sequencing

RNA-sequencing was performed on high quality (RIN > 8.0) DNA-free RNA in three batches, a larger batch contained 48 samples describing in triplicate the control 54-6 and FSHD 54-12 cell lines at eight time points during differentiation: 0 min (confluent proliferating myoblasts), 440 min (control 54-6 alignment), 530 min (FSHD 54-12 alignment), 1355 min (control 54-6 initiation of fusion), 1505 min (FSHD 54-12 initiation of fusion), 1860 min (control 54-6 myotube formation), 2165 min (FSHD 54-12 myotube formation) and 5040 min (myotube maturation). A second batch was performed for validation and consisted of 24 samples describing in triplicate the 16Abic, 12Abic, 16Ubic and 12Ubic cell lines at two time points: 0 min (confluent proliferating myoblasts) and 5040 min (myotube maturation). A third batch was similarly performed for validation and consisted of 18 samples describing in triplicate the 54-2, 54-A5 and 54-A10 cell lines at the same two time points of 0 min and 5040 min. For all batches, 312 000 cells were plated in 12 well plates and incubated for 48 h at 37^o^C and 5% CO_2_. The 0 min sample was then harvested with cells in proliferation medium, before switching sister cultures to differentiation medium and harvesting after 5040 min.

RNA was isolated using miRNeasy kit including DNase digestion (Qiagen, Manchester, UK) from each cell line at each time point in triplicate. RNA quality/concentration were checked by LabChip Bioanalyzer and Nanodrop and RNA-seq libraries were prepared using the Agilent SureSelect stranded RNAseq protocol, which allows polyA selection but was modified to work with ribo-depletion. Libraries were sequenced on an Illumina HiSeq2500. Raw reads were trimmed using trim-galore, utilising cutadapt14 (v0.4.0) to remove the Illumina Sequencing Adapter (AGATCGGAAGAGC) at the 3′ end. Additionally, 12 bases were also trimmed from the 5′ end of the reads since they showed a biased distribution. Reads were mapped to the human transcriptome using the human genome sequence GRCh38 and v82 gene annotations downloaded from Ensembl. Mapping was performed using tophat 15 (v2.1.0) and bowtie 16 (v1.1.0), enabling the fr-firststrand option of tophat to restrict mapping to the sense strand of the transcript. Reads were assigned to genes using the featureCounts program 17 (v1.5.0), counting fragments and ignoring multi-mapping reads and restricted to the sense strand. The resulting matrix of read counts was analysed using R. RNA-seq data are available from the GEO data base (https://www.ncbi.nlm.nih.gov/geo/), accession numbers GSE102812 and GSE123468.

### Transcriptomic analysis

The three batches were internally controlled and hence analysed separately as independent data sets. Each set was normalised using the DESeq package in R. Principal component analysis revealed tight clustering of triplicates within each batch. For both data sets, the dominant principal component associated with myogenic differentiation time and ordered samples by progression of differentiation, regardless of cell type. The second principal component associated with FSHD status. Hence, the two dominant components of variability of our data associate directly with the variables of interest.

For the larger data set, a multivariate regression approach was employed to analyse how gene expression varied with myogenic differentiation and cell type, an interaction term was also included to determine how gene expression dynamics over differentiation time was influenced by cell type. The regression model for each gene *i* was as follows:

$$ {E}_i={a}_i{FSHD}_{status}+{b}_i{Time}_{differentiation}+{c}_i\left({FSHD}_{status}:{Time}_{differentiation}\right) $$

For each gene, the model was fit and the significance of the positivity or negativity of each coefficient was determined at the 5% level. The genes corresponding to the top 500 positive and negative coefficient values were selected for GSEA, which was performed using a Fisher’s exact test against the gene sets described by the Molecular Signatures Database (39).

For evaluation of the expression of 500 genes found most significantly dynamically repressed in FSHD, we obtained the average of these genes in each sample corresponding to 16Abic and 16Ubic myoblasts and myotubes and performed a *t*-test to compare mean expression across samples, significance was assessed at the 5% level. For the evaluation of the expression of *PPARGC1A* and *ESRRA* gene expression in the FSHD myoblasts (54-2, 54-A5, 16Abic and 12Abic) alongside matched controls (54-A10, 16Ubic and 12Ubic), the normalised expression levels were *z*-normalised within patient-control group and an unpaired Wilcoxon test was employed to compare FSHD cell line expression to control, with significance assessed at the 5% level.

### Immunolabelling

For immunolabelling, immortalized myoblasts were plated at 25 000 cells/well in 96 well plates and cultured for 48 h in proliferation medium, while primary myoblasts were cultured for 24 hours, before being switched to differentiation medium for 3 days. Cells were then fixed with 4% paraformaldehyde/PBS for 15 min, washed thrice with PBS, then permeabilised with 0.1% Triton/PBS for 10 min, washed thrice again with PBS then blocked in 10% goat serum (volume/volume percent) (DakoCytomation, Glostrup, Denmark) for 30 min before being incubated on a rocker overnight at 4^o^C with primary antibody against MyHC (MF-20, DSHB, IA, USA) at 1/400 in PBS supplemented with 1% goat serum (v/v). Cells were then washed thrice with PBS before being incubated at room temperature for 30 min with AlexFluor conjugated secondary antibodies (eBioscience, Hertfordshire, UK) diluted 1:400 in PBS supplemented with 1% goat serum, washed thrice again with PBS and incubated at room temperature for 10 min in 1:1000 DAPI (4′, 6-diamidino-2-phenylindole)/PBS. Samples were imaged on a Zeiss Axiovert 200M microscope using a Zeiss AxioCam HRm and AxioVision software version 4.4 (Zeiss). At least three fields were taken at 100× magnification for each well, resulting in quantification of over 500 cells per well.

### Time course imaging

Immortalised myoblast cell lines control 54-6 and FSHD 54-12 were plated in triplicate at confluency at the centre of a 96 well plate (25 000 cells per well) and induced to differentiate with a high volume of differentiation medium (350 μl/well to prevent medium evaporation during the 5 days of imaging). All remaining empty wells in the 96 well plate were filled with DMEM Glutamax to provide humidity to the culture chamber. Immediately after addition of differentiation medium cells, plates were placed into a Solent Scientific chamber at 37^o^C and 5% CO_2_. Cells were imaged using an Eclipse Ti-E Live Cell Imaging System by taking a 100× magnification, phase contrast image every 5 min from each well over a total of 5 days; this generated 1440 images per well per cell line, per repeat, resulting in a total of 8640 images.

### Image analysis

To analyse the images generated, we wrote a high-throughput image analysis software in R, using the EBImage package (36) (software provided as Supplementary Material, Supplementary File 1**)**. The software can autonomously process hundreds of high quality, large images in the order of minutes. When analysing immunolabelling, each image is first split into three channels. For determination MyHC+ve area the channel displaying MyHC was passed through a low pass filter and to remove noise and binarise the image and a size filter was applied to remove background labelling, the positive proportion of the image was then quantified as MyHC+ve area (Fig. 1A). The mean MyHC+ve area from three images per well was then used to calculate the mean ± SEM MyHC+ve area for the triplicate wells for each cell line and areas tested against control using an unpaired two-tailed *t*-test.

Image analysis software to interrogate the myogenesis time course imaging data adapted the immunolabelling software. Each image taken in the time course was passed through a low pass filter to reduce noise and binarise the image, high-intensity regions were filtered on size and morphology to remove objects not considered likely to correspond to cells and finally holes were filled in. The regions of high intensity following this thresholding typically corresponded to single cells. The eccentricity of each cell identified in each image by this thresholding was measured and the average eccentricity of the field was obtained for each 5 min time point. We thus obtained a time course of eccentricities in triplicate for each cell line. The triplicates showed a similar pattern of changes that were distinct for each cell line. Differential eccentricities between cell lines were assessed by an empirical Bayes approach and *P*-value histograms confirmed that differential eccentricities were detectable between cell lines. Non-linear regression was used to fit polynomial curves to the mean (over the triplicates) eccentricity time course, for each cell line, the turning points of the curves were determined to assess the most extremely eccentric and non-eccentric time points for each cell line separately (Fig. 2).

### RT-qPCR

Cells were plated in triplicate at 312 000 cells per well in 12 well plates, cultured for 48 h and then switched to differentiation medium (treated with biochanin A or following PGC1α siRNA-mediated knock-down) for 3 days before cells were harvested. RNA was isolated using miRNeasy kit and reverse-transcribed using the Reverse Transcription Kit with genomic DNA wipeout (Qiagen, Manchester, UK), RT-qPCR was performed on a Viia7 qPCR system (Life Technologies) with MESA Blue qPCR MasterMix Plus and ROX reference dye (Eurogentec Ltd, Hampshire, UK) using *TBP* expression as a control.

The primers used were as follows:

*ESRRA* forward: 5′-AAGACAGCAGCCCCAGTGAA-3′

*ESRRA* reverse: 5′-ACACCCAGCACCAGCACCT-3′

*PPARGC1A* forward: 5′-GTGAAATTGAGGAGTGCACAGTAAA-3′

*PPARGC1A* reverse: 5′-TCACAGGTATAACGGTAGGTAATGAAA-3′

*TBP* forward: 5′-CGGCTGTTTAACTTCGCTTC-3′

*TBP* reverse: 5′-CACACGCCAAGAAACAGTGA-3′

### siRNA knock-down

Cells were plated in triplicate at 80 000 cells per well in six well plates and incubated for 24 h before each well was transfected. PGC1α mixed or control scrambled siRNA (Qiagen, Manchester, UK, catalogue number 1027416). Solutions containing 1.5 μl of either four mixed siRNAs (10 μm) against PGC1α or control siRNA (10 μm) and 150 μl OptiMem and 9 μl RNAiMax with 150 μl OptiMem, were incubated at room temperature for 5 min before mixing, then incubated at room temperature for a further 20 min. Cells were then incubated in the mixture diluted at one-eighth in proliferation medium for 24 h at 37^o^C and 5% CO_2_, before trypsinisation and replating at a density of 25 000 cells per well in 96 well plates. Cells were cultured for 2 days before switching to differentiation medium for 3 days followed by fixation and immunolabelling.

## Supplementary Material

Supplementary Data
