# Supplementary material for: Dynamic transcriptomic analysis reveals suppression of PGC1α/ERRα drives perturbed myogenesis in facioscapulohumeral muscular dystrophy
Source: Hum Mol Genet. 2018 Dec 6;28(8):1244–59. doi: 10.1093/hmg/ddy405 (PMC6452176; doi:10.1093/hmg/ddy405)
Supplement: Supplementary Data [file suppl_ddy405.zip › Banerji et al HMG-2018 - Supplementary Material - File, Figure and Table legends.pdf]

Banerji et al. HMG - 2018

Supplementary Material: Legends for Supplementary File, Supplementary Figures and Supplementary Tables

Supplementary Material, Supplementary File 1: Image analysis software

R script for a shiny based user interface for the high-throughput image analysis software employed for processing and quantifying immunolabelling data.

Supplementary Material, Supplementary Video 1A and B: Videos of control 54-6 and FSHD 54-12 myoblasts differentiating over 5 days.

A) Representative video of control 54-6 myoblast differentiation

B) Representative video of FSHD 54-12 myoblast differentiation

Supplementary Material, Figure S1: Images of control 54-6 and FSHD 54-12 myoblasts at the 8 time points selected for RNA-sequencing by consideration of eccentricity time-course of morphological changes.

Images corresponding to each of the 8 time points selected from differentiating control 54-6 and FSHD 54-12 myoblasts for transcriptomic analysis (Magnification: x100) are displayed alongside the time-course of average eccentricity.

Supplementary Material, Figure S2: Images of control 54-6 and FSHD 54-12 myoblasts at time of harvesting for RNA-sequencing

Phase contrast images of control 54-6 and FSHD 54-12 myoblasts taken at time of harvesting the wells for RNA-sequencing, to confirm morphological staging matched that observed during the selection of the time points (Magnification: x100).

Supplementary Material, Table S1: Description of cell lines analysed

Cell line, FSHD status, Relationship to matched control cell line, Gender, Muscle Biopsied and Reference for the twelve myoblast lines investigated.

Supplementary Material, Table S2: GSEA results for genes up-regulated in FSHD

GSEA results for the top 500 genes positively associated with the co-efficient a_i in the multivariate analysis of the time-course RNA-seq data describing control 54-6 and FSHD 54-12 myoblast differentiation. These gene sets are up-regulated in FSHD.

Supplementary Material, Table S3: GSEA results for genes down-regulated in FSHD

GSEA results for the top 500 genes negatively associated with the co-efficient a_i in the multivariate analysis of the time-course RNA-seq data describing control 54-6 and FSHD 54-12 myoblast differentiation. These gene sets are down-regulated in FSHD.

Supplementary Material, Table S4: GSEA results for genes up-regulated during myogenesis

GSEA results for the top 500 genes positively associated with the co-efficient b_i in the multivariate analysis of the time-course RNA-seq data describing control 54-6 and FSHD 54-12 myoblast differentiation. These gene sets are up-regulated during myogenesis.

Supplementary Material, Table S5: GSEA results for genes down-regulated during myogenesis

GSEA results for the top 500 genes negatively associated with the co-efficient b_i in the multivariate analysis of the time-course RNA-seq data describing control 54-6 and FSHD 54-12 myoblast differentiation. These gene sets are down-regulated during myogenesis.

Supplementary Material, Table S6: GSEA results for genes up-regulated during FSHD myogenesis

GSEA results for the top 500 genes positively associated with the co-efficient c_i in the multivariate analysis of the time-course RNA-seq data describing control 54-6 and FSHD 54-12 myoblast differentiation. These gene sets are up-regulated specifically during FSHD myogenesis.

Supplementary Material, Table S7: GSEA results for genes down-regulated during FSHD myogenesis

GSEA results for the top 500 genes negatively associated with the co-efficient c_i in the multivariate analysis of the time-course RNA-seq data describing control 54-6 and FSHD 54-12 myoblast differentiation. These gene sets are down-regulated specifically during FSHD myogenesis.
