# Supplementary material for: Dynamic transcriptomic analysis reveals suppression of PGC1α/ERRα drives perturbed myogenesis in facioscapulohumeral muscular dystrophy
Source: Hum Mol Genet. 2018 Dec 6;28(8):1244–59. doi: 10.1093/hmg/ddy405 (PMC6452176; doi:10.1093/hmg/ddy405)
Supplement: Supplementary Data [file suppl_ddy405.zip › Banerji et al HMG-2018-D-00584 Supplementary File 1.pdf]

```
rm(list=ls())
library(shiny)
library(shinyFiles)
library('EBImage')
stain_col<-function(x){if(x=="green"){return(2)}else{return(1)}}
setwd("~/")

server<-shinyServer(function(input, output, session) {

  # dir
  shinyDirChoose(input, 'dir', roots = c(home = '~'), filetypes =
c('', 'txt'))
  dir <- reactive(input$dir)
  output$dir <- renderPrint(dir())

  # path
  path <- reactive({
    home <- normalizePath("~")
    file.path(home, paste(unlist(dir()$path[-1]), collapse
= .Platform$file.sep))
  })

  # files
  output$files <- renderPrint(list.files(path()))
  #add in directories
  dirs<-reactive({
    list.files(path=path(),all.files = TRUE,full.names = TRUE,
recursive = TRUE)->f
    f[grep(input$suffix,f,fixed=T)]->fn
    unlist(fn)
    })
  output$dirs <- renderPrint(dirs())
  #####
  #####
  test<-reactive({
    if(input$test=="No"){progress<-"Awaiting start instructions"}
else if(input$test=="Yes Please"){m_file_dir<-dirs()[1]
    readImage(files=as.character(m_file_dir),type=input$file_t)->p
    colorMode(p)=Grayscale

thresh(p[,,stain_col(input$MYH_c)],w=220,h=220,offset=input$threshol
d)->p_t
    display(p_t)}
  })
  output$thresh<-renderPrint(test())
  result<-reactive({
    if(input$start=="Not Yet!"){progress<-"Awaiting start
instructions"}else if(input$start=="Just First Image"){m_file_dir<-
dirs()[1]
  ####
  readImage(files=as.character(m_file_dir),type=input$file_t)->p
  colorMode(p)=Grayscale

thresh(p[,,stain_col(input$MYH_c)],w=220,h=220,offset=input$threshol
```

```
d)->p_t
  p_t->MYH
  bwlabel(p_t)->p_f_l
  computeFeatures.shape(p_f_l)->f
  readImage(files=as.character(m_file_dir),type=input$file_t)->merge
  sum(f[,1])/(dim(p_f_l)[1]*dim(p_f_l)[2])*100->per_MYH
  thresh(p[,,3],w=20,h=20,offset=0.02)->p_t
  opening(p_t,makeBrush(5,shape="disc"))->p_t_o
  fillHull(p_t_o)->p_fill
  bwlabel(p_fill)->p_f_l
  MYH->m
  gblur(m,sigma=8)->m
  which(p_fill==0)->dap
  m[dap]<-0
  thresh(m,w=40,h=40,offset=0.1)->m_t
  opening(m_t,makeBrush(3,shape="disc"))->m_t_o
  fillHull(m_t_o)->m_fill
  which(m_fill==0)->dap2
  m[dap2]<-0
  bwlabel(m)->m_l2
  res = paintObjects(m_l2, merge, col=c("white","green"))
  res = paintObjects(MYH, res, col="pink")
  if(input$imo=="Yes Please"){writeImage(res,file=paste("anno",
1),type="jpg")}
#  display(res)
  computeFeatures.shape(p_f_l)->f_d
  computeFeatures.shape(m_l2)->f_m
  sum(f_m[,1])/sum(f_d[,1])*100->per_FUS
  print(dirs()[1])
  cat('% Area MYH+=', per_MYH,"%",'\n')
  cat('% Fusion=', per_FUS,"%",'\n')
    #c(as.character(dirs()[1]),per_MYH,per_FUS)
  }
    else if(input$start=="DO IT!! (All images)"){mapply(function(j){
      m_file_dir<-dirs()[j]
      ####
      readImage(files=as.character(m_file_dir),type=input$file_t)->p
      colorMode(p)=Grayscale

thresh(p[,,stain_col(input$MYH_c)],w=220,h=220,offset=input$threshol
d)->p_t
      p_t->MYH
      bwlabel(p_t)->p_f_l
      computeFeatures.shape(p_f_l)->f
      readImage(files=as.character(m_file_dir),type=input$file_t)-
>merge
      sum(f[,1])/(dim(p_f_l)[1]*dim(p_f_l)[2])*100->per_MYH
      thresh(p[,,3],w=20,h=20,offset=0.02)->p_t
      opening(p_t,makeBrush(5,shape="disc"))->p_t_o
      fillHull(p_t_o)->p_fill
      bwlabel(p_fill)->p_f_l
      MYH->m
      gblur(m,sigma=8)->m
      which(p_fill==0)->dap
```

```
      m[dap]<-0
      thresh(m,w=40,h=40,offset=0.1)->m_t
      opening(m_t,makeBrush(3,shape="disc"))->m_t_o
      fillHull(m_t_o)->m_fill
      which(m_fill==0)->dap2
      m[dap2]<-0
      bwlabel(m)->m_l2
      res = paintObjects(m_l2, merge, col=c("white","green"))
      res = paintObjects(MYH, res, col="pink")
      if(input$imo=="Yes Please")
{writeImage(res,file=paste("anno",j),type="jpg")}
 #     display(res)
      computeFeatures.shape(p_f_l)->f_d
      computeFeatures.shape(m_l2)->f_m
      sum(f_m[,1])/sum(f_d[,1])*100->per_FUS
      print(dirs()[1])
      cat('% Area MYH+=', per_MYH,"%",'\n')
      cat('% Fusion=', per_FUS,"%",'\n')
      return(c(as.character(dirs()[j]),per_MYH,per_FUS))},
1:length(dirs()))->results
      t(results)->r
      colnames(r)<-c("file name","% MYH area","% Fusion")
      write.csv(r,file=input$res)
      }

  })
  output$progress <- renderPrint(progress())
  output$result<-renderPrint(result())
  })


ui<-shinyUI(fluidPage(sidebarLayout(

  sidebarPanel(
    shinyDirButton("dir", "Chose directory", "Upload"),
    selectInput("file_t", "Image File Type:",
                choices=c("jpg","tiff","png")),
    selectInput("MYH_c", "MyHC Stain Colour:",
                choices=c("green","red")),
    textInput("suffix", "Merged Image Suffix:", "(DAPI+FITC).JPG"),
    sliderInput("threshold", "MyHC Background Threshold",
                min = 0.001, max = 0.1, value = 0.002, step= 0.001),
    textInput("res", "Results File Name", "Results.csv"),
    selectInput("imo", "Output annotated images?",
                choices=c("Yes Please","No")),
    selectInput("test", "Test Threshold",
                choices=c("No","Yes Please")),
    selectInput("start", "Start Run?",
                choices=c("Not yet!","Just First Image","DO IT!!
(All images)"))
  ),
    mainPanel(
    h4("Results"),
    verbatimTextOutput("result"),br(),
```

```
    h4("Image Files"),
    verbatimTextOutput("dirs"),
    h4("Thresh"),
    verbatimTextOutput("thresh")
  )

)))


shinyApp(ui = ui, server = server)->sa
runApp(sa)
display()
```
