# Supplementary figures and images for: Dynamic transcriptomic analysis reveals suppression of PGC1α/ERRα drives perturbed myogenesis in facioscapulohumeral muscular dystrophy
Source: Hum Mol Genet. 2018 Dec 6;28(8):1244–59. doi: 10.1093/hmg/ddy405 (PMC6452176; doi:10.1093/hmg/ddy405)

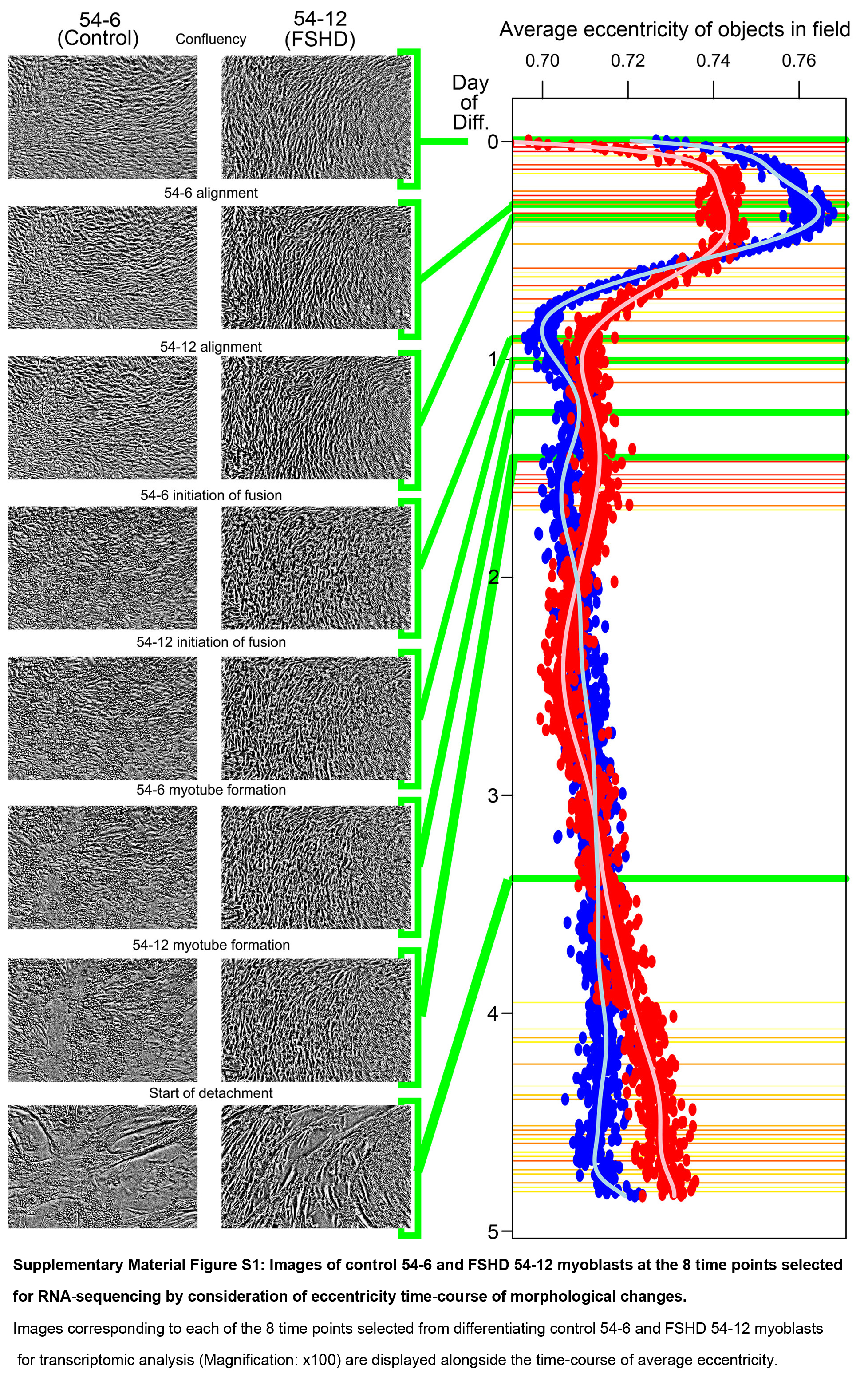

Supplement: Supplementary Data [file suppl_ddy405.zip › Banerji et al HMG-2018 Supplementary Material Figure S1.jpg]

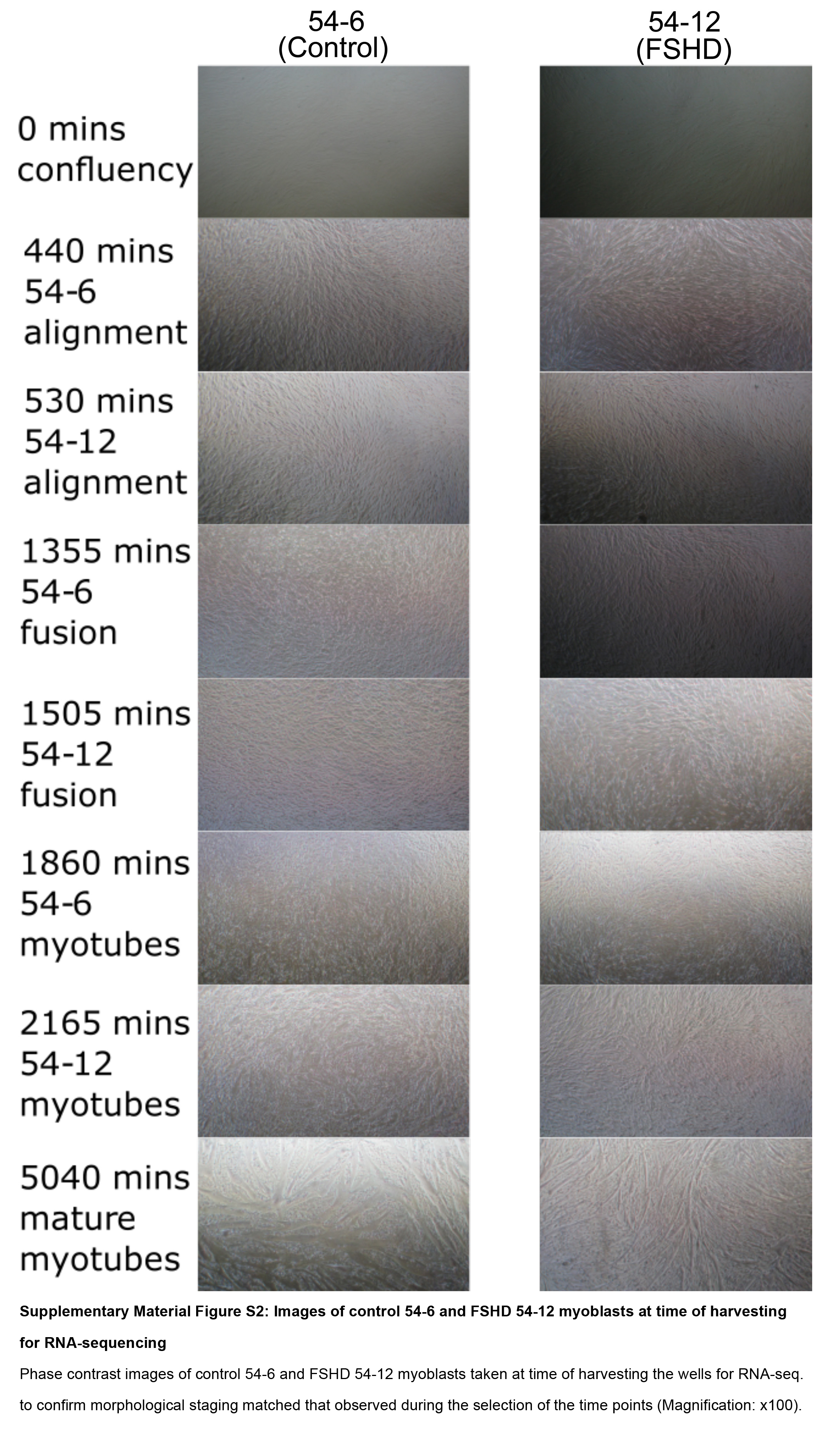

Supplement: Supplementary Data [file suppl_ddy405.zip › Banerji et al HMG-2018- Supplementary Material Figure S2.jpg]
